# Accountability strategies for sexual and reproductive health and reproductive rights in humanitarian settings: a scoping review

**DOI:** 10.1186/s13031-020-00264-2

**Published:** 2020-04-07

**Authors:** Marta Schaaf, Victoria Boydell, Mallory C. Sheff, Christina Kay, Fatemeh Torabi, Rajat Khosla

**Affiliations:** 1 Heilbrunn Department of Population and Family Health, Mailman School of Public Health, Columbia University, 60 Haven Avenue, B2, New York, NY 10032 USA; 2 Global Health Centre, Geneva Graduate Institute, Maison de la paix, Rue Eugene-Rigot 2, 1211 Geneva, Switzerland; 3 Maison de la paix, Rue Eugène-Rigot 2, 1211 Geneva, Switzerland; 4 Department of Demography, University of Tehran, Tehran, Iran; 5 Family, Women’s and Children’s Health, World Health Organization, avenue Appia 20, 1211, 27 Geneva, Switzerland

**Keywords:** Sexual and reproductive health, Reproductive rights, Accountability, Governance, Humanitarian/ development nexus, Humanitarian

## Abstract

**Background:**

Many of the 35 million women and girls aged 15–49 requiring humanitarian assistance have inadequate access to the sexual and reproductive health (SRH) services to which they are entitled. Ensuring accountability is critical to realizing their SRH and reproductive rights (RR).

**Objectives:**

This scoping review examines the extent and nature of existing evidence on accountability strategies for SRH in humanitarian settings in different geographical scopes/contexts, and contextualizes these findings in the larger thematic literature. This review seeks to answer the following questions: What accountability strategies are employed to address the availability, accessibility, acceptability, and quality of SRH in humanitarian settings? What do we know about the successes and challenges of the given strategies? What are the implications for practice?

**Methods:**

We consulted public health, social science, and legal databases including SCOPUS, PubMed, ProQuest, and LexisNexis for peer-reviewed articles, as well as Google Advanced search for grey literature; the search was conducted in March 2019. We searched for relevant articles and documents relating to accountability, humanitarian, and SRH and/or RR. To identify key challenges not reflected in the literature and additional grey literature, 18 key informants from international NGOs, local government bodies, academia, and donor agencies were interviewed from March–June 2019.

**Results:**

A total of 209 papers and documents were identified via our literature searches and interviews for review. We identified three categories of approaches to accountability in our background reading, and we then applied these to the papers reviewed a priori*.* We created a fourth category based on our findings. The categories include: (1) humanitarian principles, codes of conduct, and legal instruments; (2) technical, performance, and impact standards; (3) efforts to solicit and address the rights and needs of the affected populations, or “listening and responding,” and, (4) accountability demands made by affected populations themselves. Almost all papers identified referred to challenges to realizing accountability in humanitarian contexts. There are promising accountability approaches – some specific to SRH and some not - such as open-ended feedback from affected populations, quality improvement, and practical application of standards. Reflecting a largely top down orientation, papers concentrate on accountability mechanisms within humanitarian work, with much less focus on supporting affected populations to deepen their understanding of structural causes of their position, understand their entitlements, or access justice.

**Conclusion:**

In the last 20 years, there has been increasing standard and guideline development and program experiences related to accountability in humanitarian settings. Yet, the emphasis is on tools or mechanisms for accountability with less attention to changing norms regarding SRH and RR within affected communities, and to a lesser extent, among implementers of humanitarian programs or to institutionalizing community participation.

## Background

At the start of 2019, over 70.8 million people lived in areas affected by conflict [1]. Conflict situations are increasingly protracted, with the average time spent in displacement now estimated at 26 years [2]. The number of people affected by natural disaster grows with accelerating climate change, with 17.2 million people affected in 2018 [1]. In 2019, UNFPA estimated that out of the 100 million people requiring humanitarian assistance for reasons related to conflict and natural disasters, 35 million were women and girls aged 15–49 [3]. Inadequate access to sexual and reproductive health (SRH) services during emergencies contributes to unintended pregnancies, unsafe abortion, maternal morbidity and mortality, and increased incidence of sexually transmitted infections [4]. Moreover, major social disruption and heightened insecurity feed potentially high rates of Sexual and Gender-Based Violence (SGBV), including Sexual Exploitation and Abuse (SEA) [5]. Women and girls are uniquely affected by maternal health concerns and disproportionately affected by SGBV. However, men and boys also have SRH needs, and can be victims of SGBV [4].

Following failures in the humanitarian response to the 1994 genocide in Rwanda, the 2004 tsunami in Asia, and the 2010 earthquake in Haiti, as well as well-publicized examples of sexual exploitation and abuse perpetrated by humanitarian workers, global level platforms, guidelines, and frameworks to support operational changes to promote accountability in humanitarian settings have mushroomed [6, 7]. At the same time, in the development field, funding and research related to accountability for public services, including health, are burgeoning [8]. Relatedly, the global health establishment acknowledges that ongoing gaps in health care quality undermine the effectiveness of increasing health care coverage. Strategies and programs to improve quality draw attention to factors germane to accountability, such as respectful care, corruption, and supervision [9]. SRH and RR are differently complex than other areas of accountability work because of their intimate nature and relationship to deeply seated norms. Realizing accountability in these domains may require shifting social, religious, and political values relating to gender, reproduction, and power among affected populations, public and private sector providers, and humanitarian workers [10].

Accountability is mainstreamed into global health and human rights governance mechanisms that guide donor and government action. Sustainable Development Goals 3, 5 and 16 mandate improvements in health, the rights of women and girls, and access to justice; their constituent indicators provide a monitoring framework for governmental accountability. Multi-stakeholder strategies guiding investment in the SDGs emphasize accountability for realizing sexual and reproductive health (SRH) and reproductive rights (RR), as well as the other SDGs. The United Nations (UN) Secretary General’s updated Global Strategy on Women’s, Children’s, and Adolescents’ Health and the associated Independent Accountability Panel; and the World Health Organization (WHO) and Office of the High Commissioner of Human Rights, High Level Working Group on Health and Human Rights foreground accountability as both a strategy and a desired outcome [11, 12].

This paper is a scoping review synthesizing conceptual approaches, implementation strategies, and programmatic lessons in the rapidly growing field of accountability in humanitarian contexts, specifically for a rights-based approach to SRH. We examine the extent and nature of existing evidence on accountability strategies for SRH in humanitarian settings in low and middle income countries, and contextualize these findings in the larger literature on accountability in humanitarian settings. This review seeks to answer the following questions: What accountability strategies are employed to address the availability, accessibility, acceptability, and quality of SRH in humanitarian settings? What do we know about the successes and challenges of the given strategies? What are the implications for practice?

We define accountability as encompassing answerability and sanctions [13] regarding the realization of the right to health. Answerability entails the obligation to answer questions about decisions and/or actions [13, 14], and sanctions entail some kind of negative outcome – formally or informally levied - for failures and transgressions [13]. The international human rights community stresses the remedy component of accountability, including, for example, restitution, which seeks to restore someone whose rights are violated to their position before the violations occurred; compensation, or a financial award for harms incurred; and guarantees of non-repetition [15, 16].

## Methodology

In this scoping review, we address accountability of health, social, and judicial service providers and those who regulate them, to the putative recipients of these services, namely refugees, stateless or internally displaced people (IDPs), people with temporary protection, and host country populations. We refer to these service recipients as “affected populations” for the rest of the paper, unless greater specificity is required.

We chose to undertake a scoping review as this allowed us to assess a range of evidence from varying streams of literature, and to employ diverse study selection approaches. The review adopts Colquhoun et al’s approach to scoping reviews, which takes into account the fact that researchers develop increased familiarity with the subject matter and available literature through the data collection process, and thus allows for an iterative research process through the use of post-hoc inclusion and exclusion criteria [17–19]. Data were collected through a search of peer-reviewed and grey literature and through key informant interviews. These key informant interviews provided complementary information on challenges to accountability and confirmation of emerging findings, and helped us to identify additional grey literature materials to be included in our review.

### Literature search

To identify peer-reviewed literature, we consulted public health, social science, and legal databases (Embase, SCOPUS, PubMed, ProQuest, and Lexis Nexus) in March 2019. We searched for relevant articles and documents using three groups of search terms, relating to: 1) accountability, 2) humanitarian, and 3) SRH and/or RR. For each, an extensive list of synonyms and subcomponents were compiled by MS and VB. For example, “family planning,” and “cervical cancer” were part of the SRH group, “disaster” and “conflict” were part of the humanitarian group, and “grievance redress” and “community score cards” were part of the accountability group. All searches included terms from at least the humanitarian and accountability group, so all results related to accountability in humanitarian settings, and many related to accountability for SRH and/or RR in humanitarian settings. Example searches are provided in Table 1. The peer-reviewed literature search was conducted by CK.

**Table 1:**
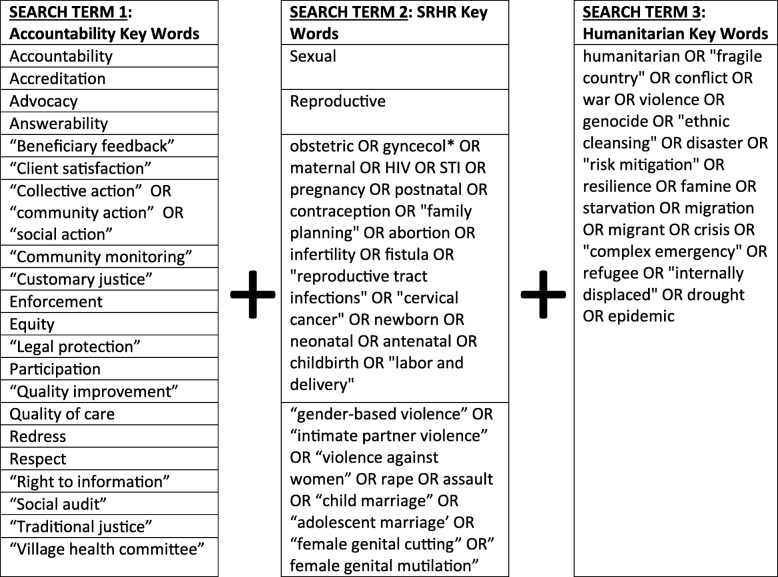
Example of search terms.

To search the grey literature, we applied the same combination of search terms in Google Advanced search, limited to PDFs and to publications in the English language. Google results were reviewed until there were five results in a row that were not relevant to the scope. The grey literature search was conducted by VB.

Articles, including studies, books, book chapters, and reports published in English and in French from January 1, 2009 to March 30, 2019 were selected for the review. We included papers related to SRH and RR of any gender in our review, though the overwhelming majority of papers focused on women and/or girls. We included papers that addressed gender, health, and humanitarian contexts, but excluded papers that were just about gender and humanitarian contexts.

Davis identifies three broad approaches to promoting accountability in the humanitarian system, including (1) the development and use of legal instruments, humanitarian principles, and codes of conduct; (2) the development and use of technical, performance, and impact standards; and, (3) “a focus on the rights and needs” of the “claimants” of humanitarian assistance, which may be realized through participatory efforts, context-sensitive programming, and mechanisms for “listening and responding” [20]. This was the broadest categorization we identified in our background reading. Given that we aimed to produce a broad scoping review, we used Davis’ categories to organize our findings. We adjusted these categories to reflect the actual content of the papers identified by narrowing the third category to “listening and responding,” and adding a fourth category of “accountability demands made by affected populations.”

Based on title and cursory review of the abstract, we identified a total of 288 papers and documents for review: 176 papers in the peer reviewed health and social science journals, 53 papers in the grey literature, and 24 papers in legal databases. We identified an additional 35 frameworks and guidelines via recommendations from the key informant interviews and hand searching stemming from reviewed articles. The papers were exported into *Zotero*, an open-source reference management software, and duplicates were removed. All the titles, abstracts and summaries were reviewed (authors MS and VB) against the inclusion criteria. Table 2 details the inclusion criteria.

**Table 2:** Inclusion/ Exclusion Criteria

Criteria	Inclusion	Exclusion
*Article Type*	Peer- reviewed studies, book chapters, book, reports Grey literature in PDF	
*Location*	Humanitarian crises (armed conflict, famine, and natural disaster) in a low or middle income country, as defined by the World Bank	Middle income countries with disease outbreak only (e.g.- Zika in Brazil)
*Date*	January 2009–March 2019	Anything before 2009
*Language*	English and French for articles, English only for grey literature	Any other language
*Population of interest*	Populations affected by humanitarian crises (refugee, internally displaced, host country population)	
*Key themes*	• Accountability: as described by Davis, 2007 • SRH, as defined by WHO in 2004 [21]	Focus on disease outbreaks that impact SRH with no relation to humanitarian settings; no mention of accountability or closely related themes (e.g. transparency or governance)

The papers were reviewed in full, and a detailed data extraction in Excel was undertaken for SRH in humanitarian contexts (authors MCS, CK, and FT) and accountability projects directly related to SRH in humanitarian settings (authors MS and VB). The extraction tables contained fields relevant to our review, including strategy or program description, key findings, challenges, facilitators, and relevant contextual considerations. Law review articles and papers describing the wider landscape of accountability in humanitarian settings provide important contextual information, but could not fit into the extraction tool. Thus, these papers were coded (authors MS and VB) using a codebook with deductive thematic and inductive emergent codes. The codes were descriptive rather than interpretive. The most frequently used codes included: “feedback mechanisms,” “history/evolution of accountability in humanitarian settings,” “barriers to accountability in humanitarian settings,” “access to justice,” “SEA,” “GBV,” and “engagement of local civil society.” A full list of codes is included as Additional file 1.

### Key informant interviews

Eighteen key informant interviews were conducted with experts and practitioners working in the humanitarian sector focused on accountability, SRH, and/or RR. Eleven respondents worked in international NGOs, two worked in multi-lateral organizations, one worked with a government body in a humanitarian context, one worked in an international donor agency, and three worked in academia. The majority of the interviewees had significant recent field experience. These respondents were selected based on our knowledge of key experts and practitioners, guidance from the Human Reproduction Program at the World Health Organization, and snowball sampling. Respondents were recruited using an introductory email explaining the purpose of the review. Four (MS, VB, CK, MCS) authors conducted the interviews using semi-structured interview guides that allowed for probing regarding emergent findings from the literature reviews. The objective of the interviews was to confirm and explore the “field relevance” of emerging findings, identify new grey literature materials, and discuss challenges to accountability in humanitarian settings; therefore, it was not necessary to record or transcribe the interviews. The interviews informed our search and added nuance to our analysis, and did not aim to explore the lived experience of the interviewee or other more complex phenomena typical of qualitative research and analysis. Thus, the interviewers took detailed notes, which were then coded by VB using the same codes developed for the context and law papers, and summarized and integrated with the other results. Reports and other documents suggested by the interviewees were added to the literature review for additional contextual and project-specific information.

The scoping review received an exempt determination status from the Columbia University Medical Center ethical review board, as interviewees were asked to speak in their professional capacity.

### Data synthesis

In line with scoping review objectives of synthesizing and categorizing the existing literature on a given topic, we first summarized each extraction table to represent the general findings of each theme. MCS, CK, and FT completed this task, with direction from MS and VB. Second, following conversation with RK, MS and VB grouped the thematic and emergent codes we had applied to the law review, wider humanitarian context, and interview transcripts into broader groups and synthesized the findings. We assessed the main features of the included data, including key trends, emerging issues and challenges, and similarities and discrepancies. These syntheses and summaries form the basis of the results section.

The ultimate number of sources cited differs from the total number included in our analysis, as we added some sources while we wrote the paper (for example, we wanted further information on an initiative described in an article), and as some of the papers appear only in the extraction tables but are not cited in the text. Finally, some papers were included in our synthesis but were not included in this manuscript, such as those discussing more tangential issues like discourse and media presentations of humanitarian settings, or, those grey literature sources that merely summarized the studies we had already included. We do summarize and cite 100 % of the papers that likely would have been identified in a systematic review – the 27 papers that we identified focusing explicitly on interventions to promote accountability for SRH and/or RR.

## Results

After reviewing the 288 papers and documents (176 from the peer-reviewed literature; 53 from the grey literature; and 24 from legal databases), 114 papers were excluded, leaving a total of 174. After including the additional 35 papers identified during the key informant interviews, a total of 209 articles were assessed (Table 3). These 209 papers and documents were grouped into five categories: (1) projects explicitly aiming to promote accountability for SRH in humanitarian settings (see Additional file 2); (2) SRH in humanitarian contexts, with at least some discussion of accountability or closely related themes; (3) law review articles; (4) papers describing the wider context of accountability in humanitarian settings; and, (5) relevant frameworks and guidelines.

**Table 3:** Number of Papers per Category

Thematic Area	Final included
SRH in humanitarian contexts, with at least some discussion of accountability or closely related themes;	73
Projects explicitly aiming to promote accountability for SRH in humanitarian settings	27
Law review articles	14
Papers describing the wider context of accountability in humanitarian settings	60
Frameworks/guidelines	35
**Total**	**209**

The results are presented in two sections. The first section describes four prevalent strategies for accountability in humanitarian settings, including the 27 papers that describe specific interventions for accountability for SRH and RR (see Additional file 2). We flesh out our synthesis of these 27 papers with insights and context from 48 other papers reviewed from public health, the social sciences, the law, and grey literature, as well as from interviews [22–70]. The second section summarizes challenges for accountability in humanitarian settings detailed in the papers reviewed and in the interviews.

### Approaches to promoting accountability in humanitarian settings, with particular consideration for SRH and RR

We used Davis’ three categories of accountability to organize our findings, and, based on what emerged in our literature search, we added another category of approaches to promoting accountability: those driven by affected populations themselves [20]. A summary of these strategies is provided in Table 4.

**Table 4:** Summary of key accountability strategies for SRH in humanitarian settings

Key Accountability Strategy	***Key elements of the strategy***
The development and use of humanitarian principles, codes of conduct, and legal instruments	*Principles and codes of conduct*: Fundamental principles and codes of conduct developed by international NGOs, UN agencies, and other international humanitarian and development stakeholders serve as a guide for expected behavior. There are 3 somewhat distinct streams of principles and codes relating to: • 1) UN commitments to preventing and addressing GBV, and women’s peace and security, including sexual violence in conflict; o Example: UN resolution 1325, which recognized rape and sexual violence as a war crime and provided an organizing framework for the creation of specific mechanisms to prevent violations and to prosecute perpetrators • 2) UN commitments to ending SEA perpetrated by UN peacekeepers or other humanitarian workers; and, o Example: UN Secretary General’s Bulletin on SEA, which defined SEA and the duties of UN personnel to prevent and address SEA • 3) NGO-led processes for accountability for service delivery and protection of affected populations; NGOs formed organizations that elaborated quality and/or accountability standards and supported members to meet those standards o Examples: People in Aid, SPHERE, Humanitarian Accountability Partnership, Core Humanitarian Standard Alliance
	*Legal Instruments and access to justice*: Both formal and hybrid (formal and informal) instruments have been developed by host countries, international NGOs, UN agencies, and affected populations themselves to support affected population access to justice for SRH, primarily in camp settings. These include creating legal aid programs, training the judiciary, supporting individuals to pursue cases, and raising awareness among affected populations about their rights and entitlements. Hybrid approaches combine formal and informal community structures in community-based complaints mechanisms. Such mechanisms require community engagement in development and implementation, and include follow up and remedy mechanisms.
The development and use of technical, performance, and impact standards	UN agencies and NGOs developed technical, performance, and impact standards as a means to assess the quantity and quality of care provided to affected populations in humanitarian settings and promote accountability on SRH and RR services provided. • Examples: MISP, scorecards, needs assessment tools, tools to monitor the operationalization of standards, and quality improvement
“Listening and responding”	Humanitarian agencies created mostly close-ended and some open-ended feedback mechanisms that focus on the rights and needs of affected populations to participate and provide input on their SRH and RR needs. • Examples: community help desks, feedback boxes, community meetings, call centers, face to face or unified (group) feedback, participatory action research, and community scorecards.
Accountability demands made by affected populations	*Local women’s groups/civil society* Women’s groups and other civil society organizations advocated for their self-identified needs and demands, ranging from participation in program planning to protesting their exclusion from these processes.
	*Local development and use of dispute resolution mechanisms* These strategies are typically informed by customary (not codified) law, and focus on compensation and conciliation.

#### The development and use of humanitarian principles, codes of conduct, and legal instruments

##### Principles and codes of conduct

Concerted focus on accountability within the humanitarian sector accelerated significantly after the 1996 publication of the Joint Evaluation on the Humanitarian Response in Rwanda. Among other findings, the evaluation recommended that NGOs establish self-regulation mechanisms or subject themselves to binding processes; these mechanisms should create obligations of conduct and of result [22].

The principles and codes of conduct that have developed subsequently do not reflect a linear process of norm development. Different actors had different approaches to accountability, with, for example, some suggesting voluntary codes and others stipulating binding frameworks with mechanisms for enforcement. Some norms have been updated or changed to reflect lessons learned; others have been combined, harmonized or abandoned. Some norm development processes were led by UN agencies; others were spearheaded by NGOs. There are three somewhat distinct streams of principles and codes relating to: 1) UN commitments to preventing and addressing GBV, including sexual violence in conflict; 2) UN commitments to ending SEA perpetrated by UN peacekeepers or other humanitarian workers; and, 3) NGO-led processes for accountability for service delivery and protection of affected populations. Table 5 provides a chronology of key norm and principle development in the humanitarian sector.

**Table 5:** Timeline of key accountability norms, principles, and codes of conduct*

• 1997: Sphere project launched.	
• 2003: Humanitarian Accountability Partnership (HAP) launched and quality and accountability standards start to be developed through engagement with many humanitarian agencies and affected people.	
• 2003: The HAP Standard in Accountability and Quality Management and its corresponding certification scheme was launched.	
• 2003: UN Secretary-General’s Bulletin was issued, outlining a zero-tolerance policy toward SEA, and obliging UN staff to report incidents of abuse. The Bulletin is binding on all UN staff, including all agencies and individuals who have cooperative agreements with the UN.	
• 2005: Groupe URD, an independent think tank, launched the Quality COMPASS, which contains 12 quality criteria.	
• 2006: Inter-Agency Standing Committee (IASC) statement of Commitment Eliminating SEA by UN and Non- UN Personnel, which broadened the international commitment to fight SEA by establishing standards of conduct that are applicable to all personnel at all times, including when off duty and on leave.	
• 2011: “The Transformative Agenda” was finalizedby the IASC principals, with a focus on improved leadership, coordination and accountability. An IASC task force developed Commitments on Accountability to Affected Populations (AAP); the larger IASC then agreed to incorporate these commitments into policies and operational guidelines, and to promote them within the clusters and humanitarian country teams.	
• 2012: The IASC Task Force on Accountability to Affected Populations was established to support the implementation of the AAP commitments across the humanitarian field.	
• 2012: Ground Truth Solutions, an international NGO, was founded to independently gather feedback from crisis- affected people to share with humanitarian agencies and the sector at large.	
• 2012: IASC Minimum Operating Standards for the prevention of SEA were issued, providing guidance and specific indicators on how organizations can set up internal structures to fulfil their commitments to prevent SEA.	
• 2014: The Core Humanitarian Standard (CHS) was launched, replacing HAP and the People in Aid standards; the standards were then integrated into the Sphere handbook.	
• 2014: The IASC Task Force on Accountability to Affected Populations and the Task Force on PSEA were combined.	
• 2015: People in Aid, Groupe URD, and the Humanitarian Accountability Partnership merged to become the CHS Alliance.	
• 2015: The Humanitarian Quality Assurance Initiative, an international NGO, was set up following the launch of the Core Humanitarian Standard to provide verification and certification services to NGOs seeking third party assessment of their performance against the CHS.	
• 2015: UNFPA Minimum Standards for Prevention and Response to Gender-based Violence in Emergencies was issued, to provide practical guidance on the prevention and mitigation of gender-based violence in emergencies.	
• 2016: The Grand Bargain was launched. It reflects a commitment of signatories, many humanitarian agencies and donors, to enhance local control over the humanitarian response and to improve the effectiveness humanitarian action.	
• 2017: The IASC AAP commitments were revised to align with the CHS and were fully endorsed by the executive heads of 18 United Nations and non-UN organizations that form the IASC.	
• 2018: A revised Quality & Accountability COMPASS was issued, containing a series of recommendations, processes, and tools that were specifically designed to help international aid projects implement the Core Humanitarian Standard in all sectors, contexts, and operational zones.	
• 2018: The revised Sphere Handbook was published; the principles and content of the CHS are reflected throughout the revised Handbook.	
• 2018: IASC issued revised Commitments on Accountability to Affected Populations, and the Guidance Note for Principals and Senior Managers for both the organisational and collective levels was endorsed by the IASC AAP/PSEA Task Team.	
• 2018: IASC issued a plan for Accelerating PSEA in the Humanitarian Response, with particular focus on ensuring stronger PSEA structures in countries.	

*In addition to principles and codes of conduct, this box also includes technical standards to provide readers with a full chronology of related developments

First, following concerted advocacy from women’s rights NGOs and activists, a suite of Security Council resolutions addressed issues crucial to women’s peace the security. The 2000 United Nations Security Council Resolution 1325 (S/RES/1325), recognized rape and sexual violence as a war crime and provided an organizing framework for the creation of specific mechanisms to prevent violations and to prosecute perpetrators [71]. The Security Council reaffirmed and strengthened this call for member state action and for time bound commitments to combat sexual violence through a series of subsequent resolutions [72–78].

Second, several high profile reports in the 2000s showed that SEA perpetrated by UN peacekeepers and humanitarian workers had occurred in multiple settings, instigating widespread condemnation and agreement that robust mechanisms for individual and institutional accountability were required [23–26]. The exploitation documented included the exchange of sex for goods and services or employment [26, 27]. Girls, particularly those living by themselves or heading households, were the most affected by this abuse [24, 25]. In response, the UN developed clear structures and procedures for ensuring compliance with the UN Secretary General’s Bulletin on SEA, which defined SEA and the duties of UN personnel to prevent and address SEA [28].

Third, alongside UN efforts, humanitarian and development NGOs and donors initiated their own processes of norm development in the late 1990s and early 2000s, including People in Aid, Sphere, and the Humanitarian Accountability Partnership (HAP) [29]. These are organizations comprised of NGO members that elaborated quality and/or accountability standards and supported members to meet those standards. HAP also included a certification process and produced influential Humanitarian Accountability Reports to assess and describe trends in accountability [6, 30]. In 2013, HAP, People in Aid, Sphere and others formed the Core Humanitarian Standard (CHS) Alliance, which spearheaded a lengthy participatory process to develop the Core Humanitarian Standard on Quality and Accountability. This Standard sets out nine non-sector specific commitments that organisations and individuals involved in humanitarian response apply to improve the quality and effectiveness of humanitarian assistance, including engagement and responsiveness to affected populations [30]. Sphere also partnered with other groups to launch the Humanitarian Standards Partnership, which fosters collaboration, training, and support to implement the sector specific Sphere standards [31].

The 2018 CHS Alliance Report states that implementation of principles and codes of conduct and similar mechanisms has been largely compliance-orientated and driven by headquarters, with an assumption that once employees know the rules, then behaviour change will follow [7]. We found two papers that directly examined the use and relevance of principles and codes of conduct [6, 78]. First, Matti reviewed 100 organizational websites and found that only a few did not refer to a code of conduct. However, of those that did, the code was not necessarily publicly accessible; many codes were general and not specific to the organization; and, most did not have compliance mechanisms [78]. Matti found little evidence regarding compliance with these codes of conduct and whether sanctions are levied in cases of non-compliance [78]. Second, Chynoweth’s research in two countries suggested that interpersonal accountability, or accountability imposed through shared professional norms and robust, transparent coordination platforms, may be an important complement to principles and codes of conduct, including for SRH and RR. In other words, shared norms among humanitarian workers; facilitative behaviors, such as communication, acknowledging and addressing mistakes, and constructive criticism; and rewards, can help to promote the realization of principles and codes of conduct [6].

##### Legal instruments and access to justice

Our scoping review yielded multiple sources describing how affected populations do or do not access justice in humanitarian settings. From a legal point of view, the host country has legal responsibility for the protection of refugees and IDPs, regardless of the State’s capacity to fulfil these obligations. In practice, many host States delegate their responsibilities to UNHCR, which in turn delegates aspects of these responsibilities to NGOs and other partners [32]. In camp settings, the administration of justice is undertaken by a diverse set of actors including the host government, the UN, NGOs and refugee community themselves [32, 33]. As a consequence, there are several systems of justice at play with multiple, overlapping and conflicting sources of law, including religious and customary law [34]. Customary law refers to unwritten rules that have the normative status of law [35].

Though access to justice is not explicitly recognised as an immediate basic need in the Core Humanitarian Standards, the right to remedy is essential to a human rights approach to accountability. Most of the access to justice articles we identified focused on refugee camp settings; few papers addressed the needs of non-camp refugees, IDPs, and unrecognized refugees, including those living in urban areas [34, 44, 58]. The papers focused on refugee camp settings described different ways refugees access the formal justice system, including the significant challenges they may face. The host state may lack the capacity to administer justice, may restrict refugee movement, or may be otherwise unwilling to facilitate refugees’ access to the legal system [32, 33, 36]. Financial and language barriers and poor access to transport also present challenges [32, 33, 36]. In addition, refugees may be unfamiliar with local laws, face intimidation in the host country, and fear reprisals because of lack of protection for those who make complaints or provide evidence [32, 33, 36, 37].

Three papers described efforts to increase refugee populations’ awareness of their rights to access formal and other forms of justice for SRH; they all examined a GBV program that included awareness raising and abuse reporting for refugees [79–81]. The effort was only partially successful; women did not report GBV as they were unaware of their rights, because GBV was normalized, and/or because structural discrimination against women prevented them from reporting to the male-dominated camp leadership or the customary justice system [80, 81]. Women’s wider capabilities (such as education and income) were key determinants of their ability to navigate structures for remedy, such as traditional courts or camp police systems [79].

Despite the variety of affected populations and duty bearers, we found many similarities in the descriptions of barriers to justice for all types of affected populations, particularly for GBV and SEA. Frequently cited barriers include: the application of religious and customary law reinforces gender discrimination; affected populations lack knowledge about rights; national law related to rape is inadequate; discriminatory practice by authorities; remote location of camps; fear of losing aid or other reprisals; reluctance to reveal one’s self as a victim and face stigma; lack of rights knowledge; and absence of female officers and other authorities [3, 7, 34, 38–41, 43, 80–82]. Also, some survivors may feel that exchange of sex for goods was an advantageous decision in the given circumstances [55].

In addition to undermining individuals’ right to remedy, obstacles to reporting weaken efforts to identify and estimate human rights violations for the purposes of international prosecution and for health and protection program planning [16, 38, 82]. Passive reporting is institutionalized in many settings. For example, the UN Security Council’s recommended Monitoring and Reporting Mechanism relies on survivors coming forward, likely resulting in underreporting of rape and sexual abuse [16].

Though much of the literature we identified focused on challenges, other papers described programs that improve access to the formal justice system, including for SEA and GBV. For example, the American Refugee Committee’s Legal Aid Clinic for Women operated in a long-term refugee camp. The clinics were staffed with lawyers who supported women to pursue cases in national courts [33]. Similarly, the Office of the High Commissioner of Human Rights, the United Nations Joint Human Rights Office, and local partners had a project to build the expertise of actors in the judicial system in matters of sexual violence and to encourage local judicial authority ownership of the fight against impunity for sexual violence [44]. Grey literature reports suggest that these programs can have a positive impact, but they must continuously contend with the challenges already elaborated, as well as with donor reluctance to “accrue the duty-bearing responsibility that is implied by the rights-based approach” [33].

Finally, we identified hybrid approaches that combine formal and informal community structures. Often called community-based complaints mechanisms, these approaches entail community engagement in development and implementation, and include follow up and remedy mechanisms. To best serve affected populations and to promote programmatic coherence, one unified mechanism should cover all humanitarian providers in a given locale, though multi-agency coordination is often difficult to operationalize in practice [45]. The Inter-Agency Standing Committee has created a best practice guide and standard operating procedures for community-based complaints mechanisms, based on lessons learned from pilot efforts and formal evaluations in several countries [45].

#### The development and use of technical, performance, and impact standards

The majority (14) of the 27 papers that we identified focusing explicitly on interventions to promote accountability for SRH and/or RR describe the use of tools or interventions employing technical, performance, and impact standards (see Additional file 2).

The Minimum Initial Service Package (MISP) for reproductive health (RH) is the most widely applied technical standard for SRH in humanitarian contexts. MISP components are in turn used as indicators of accountability. Developed by the Inter-Agency Working Group on Reproductive Health in Crises (IAWG) in 1995 and revised in 2019, the MISP is an agreed set of priority interventions to prevent excess morbidity and mortality at the onset of humanitarian emergencies, ensuring that service providers are immediately accountable for fulfilling certain elements of affected populations’ right to health. IAWG developed the MISP, the Inter-Agency Field Manual on Reproductive Health in Humanitarian Settings, and the Inter-Agency Reproductive Health Kits to improve the quality and coverage of SRH services in humanitarian contexts [46, 47]. The MISP is included in many of the broad accountability standards and mechanisms that have developed over the past decade, including the Sphere Humanitarian Charter, the Minimum Standards in Disaster Response, and the Inter-Agency Standing Committee Health Cluster Guide.

IAWG conducted a ten-year global evaluation of the MISP, the Inter-Agency Field Manual on Reproductive Health, and the Inter-Agency Reproductive Health Kits in 2004, and found that the quality of reproductive health services had improved for refugees in stable settings, but quality was lacking in IDP settings [46]. IAWG conducted another global evaluation in 2013–2014, and found that funding and institutional capacity for the MISP had meaningfully improved, while gaps remained in several areas, including community engagement, adolescent SRH, high quality evaluation of SRH programming, and comprehensive abortion care [47]. Onyango et al. examined available documentation on MISP implementation in five countries and found inconsistencies in the quality and completeness of the MISP activities, lack of awareness of the MISP, and no standardized monitoring [83].

Of the 14 papers identified describing the use of tools or interventions relying on technical, performance, and impact standards to promote accountability for SRH and/or RR in humanitarian contexts, two papers describe the same scorecard for assessing the provision and quality of facility services, including for SRH. The scorecard includes qualitative and quantitative components to assess service capacity, service provision, and client satisfaction, and takes 2 days to administer [84, 85]. A third paper explains how the authors adapted the WHO safe motherhood needs assessment tool to assess quality of care, including infrastructure, equipment, training and supervision, and client provider interactions [86]. The three papers described the respective tools as acceptable and effective in assessing quality of care. Challenges encountered included that response scales were not intuitive, some service providers were hesitant to respond honestly, and some service providers exhibited negative biases towards the services assessed [84–86].

Nine of the 14 papers examined projects to improve the quality of services for affected populations. The services related to maternal and neonatal health, family planning, post abortion care, and sexual violence. Interventions included a participatory quality improvement program within health facilities [87]; training staff in quality improvement or quality clinical care [48, 88] or, multi-component interventions comprised of training, supervision, supply chain improvements, or awareness raising [89–91]. All the papers reported at least some positive outcomes, from service utilization such as family planning and HIV post-exposure prophylaxis uptake [48, 87, 89–91], to improvements in health provider behavior [48], and in facility management [87, 88].

Three of the 14 papers examine the implementation and monitoring of standards. Kitabayashi describes the use of personal Maternal and Child Health (MCH) Handbooks, a client-held record intended to promote the continuum of care among women who may see multiple providers, as MCH care is fragmented and client mobility is curtailed by curfews and other limitations [92]. The handbook increased service utilization and promoted higher provider compliance with procedures, as the handbook functioned as a checklist [92]. Howard (2012) examined how SRH services contracted to non-state actors performed against predetermined benchmarks set out in the Basic Package of Health Services (BPHS); the use of these performance targets reportedly led to increased provision – and thus coverage – of reproductive health services [93].

The extent to which these efforts are embedded in larger institutional frameworks for training and supervision was key to their success [48, 83, 91]; interviewees also emphasized this finding. Institutional commitment, expressed in part through transparency about the program and manager (rather than just clinician) engagement in the project, facilitated success [48, 90, 94] as did awareness of the standards among those tasked with ensuring their implementation [83, 92, 93]. Both the MCH handbooks and the BPHS programs included activities to promote awareness and buy-in. Staff from different agencies were trained to complete the MCH handbooks [92] and accomplishment of the SRH components of the BPHS was incentivized through performance-based payments [93].

#### “Listening and responding”

Many of the new technical, performance, and impact standards and mechanisms focus on the rights and needs of affected populations to participate and provide input across all domains, not just SRH and RR. These efforts generally fall under the rubric of “accountability to affected populations” (AAP) [49]. Humanitarian agencies have created mechanisms for individual feedback, complaints, and remedy; increasing percentages of affected populations have accessed such mechanisms in the last 5 years [49–51]. In a survey of 5000 aid recipients in multiple countries conducted by the Active Learning Network for Accountability and Performance (ALNAP), those who had been able to give feedback were 3.5 times more likely to say that they had been treated with dignity and respect than those who had not been able to so [49].

Feedback mechanisms may include community help desks, feedback boxes, community meetings, and call centres [51]. Some of the attributes identified that foster the effectiveness of such mechanisms are intuitive, such as ensuring that affected populations know about the mechanism for feedback; that these mechanisms are culturally appropriate and embedded; that they are accessible to vulnerable and marginalized groups and that special outreach efforts may be required to ensure this; that agency staff at the field and management level are committed to using these mechanisms and to acting on their findings, and that they feed back this information to the community; and, that affected populations perceive the mechanism as safe, confidential, and trustworthy [49, 51, 52]. In some contexts, face-to-face feedback may be more appropriate, as the feasibility of using information and communication technology varies [52]. Also, in situations where there are multiple humanitarian agencies and providers, communication among agencies about the feedback they receive or a unified feedback mechanism may be preferable, though this requires honest, open discussion among professionals and agencies [52].

Some mechanisms have been criticized for presenting a priori questions to affected populations rather than asking them open ended questions [50], though we heard in two interviews about existing programs that do allow interviewees to share their opinions on whatever issues they chose. Many evaluations of feedback mechanisms found that data may be collected, but not used, or that input is collected only from the more powerful and privileged members of affected populations [51–53, 74]. Such feedback loop failures can have a negative impact; individuals in communities affected by a typhoon who provided feedback through information and communication technology but never received a response told researchers that they were unlikely to speak up again [43]. The CHS Alliance reports that 43 evaluations show that the two CHS commitments with the poorest performance are complaints mechanisms and communication with communities [7]. This conclusion was echoed in many peer-reviewed and grey publications, as well as in our interviews [7, 33, 41]. Moreover, sanction and remedy appear to be rarely included in these mechanisms, or at least, in their evaluations [49, 55]. In brief, mechanisms for input proliferate, but when and how these translate to meaningful influence or remedy is not known.

As further illustrated in Additional file 3, SRH and RR are explicitly addressed (or not) in reports and other key documents stemming from commitments to accountability to affected populations. Most documents do not explicitly address SRH and RR. Among those that do address SRH and RR, the following themes emerge: 1) the importance of training on SEA, which persists in humanitarian settings despite the commitment of organizations to zero tolerance and, 2) the need to collect disaggregated data in order to design appropriate programs, such as for women at different stages of pregnancy and lactation. Most documents do explicitly address gender. Among other findings, they conclude that staff with mixed genders facilitate appropriate response to communities; removing cultural and structural barriers for women’s participation increases gender equality; and a gender analysis helps to identify the unique needs and coping strategies and capacities of women, girls, boys, and men.

We identified two papers on interventions to promote the participation and influence of affected populations in SRH services in humanitarian settings [95, 96]. The first project used participatory action research [95], and the second relied on community scorecards [96]. Both profitably applied participatory approaches to support communities to raise their concerns, and facilitated dialogue and joint planning involving local health providers and community members [95, 96]. The projects fostered increased service utilization and mutual trust and understanding, which had been eroded during the Ebola epidemic or following years of governmental underinvestment of the health system [95, 96]. In the case of the community scorecard project, local level innovation and adaptation was enabled by the provision of funding for action plan implementation, and potentially by lack of central state engagement at the periphery [96]. Both interventions were adaptations of strategies that had been developed in non-humanitarian settings, suggesting that further learning and practice across the humanitarian/development continuum may be fruitful.

#### Accountability demands made by affected populations

In addition to Davis’ (2007) three broad approaches to addressing the accountability failures of the humanitarian system, our scoping review identified a fourth approach: accountability efforts driven by the affected populations themselves. We identified two types: (1) the activities of local women’s groups/civil society, and (2) local dispute resolution mechanisms/ customary justice strategies.

##### Local women’s groups/civil society

A series of papers detailed the active engagement of local grassroots women’s groups’ efforts to foment accountability for services in humanitarian settings [56–58]. Susskind (2011) describes a women’s group that was founded by and for rape survivors, which advocated for their self-identified needs and political demands [58]. Holzer (2012) authored an ethnographic article depicting actions undertaken by a women’s group in a refugee camp. Frustrated at their inability to have input, refugee women organised a series of sit-ins and strikes to protest proposed repatriation plans [56]. In another context, the camp-based Karen Women Organisation developed local solutions to increase reporting rates on GBV [57].

UN Security Council Resolution 1325 obligates states to include women’s active and meaningful participation in peace-building and in post-conflict reconstruction [97], and Resolution 1820 urges increased participation of women and calls on UN organizations to work with women-led NGOs to develop mechanisms to protect refugees from sexual violence [71]. More recently, calls to “localize” humanitarian response reflect widespread agreement among governmental and non-governmental actors on the importance of increasing local, national, and regional control over the humanitarian response, including women’s groups in particular. However, two key frameworks for localization – the Grand Bargain and the Charter for Change – include few commitments to gender, and no guidance on how to support women led organizations [60, 69]. Localization, as it is generally operationalized, does not yet entail substantive engagement of women’s organizations, or other pre-existing civil society groups, particularly in refugee situations [58, 61–63]. Several papers illustrate failures, such as a GBV sub-cluster refusal to include grassroots women’s groups in the planning and implementation of activities designed to address sexual violence in an IDP context [64]. The GBV sub-cluster meetings were held in French (rather than Creole) on a UN base far from the camps where women lived, and a difficult to obtain UN pass was required for admittance [58]. Local women’s organizations advocated nationally and internationally for more meaningful participation [58, 63].

Studies, grey literature, and interviewees pointed out that efforts to engage women and women’s groups should also consider the prevailing gender relations of power in a given context. Internalized gender norms and discomfort protesting and speaking out may contribute to silence among individual women and women’s groups, particularly when they interact with higher status individuals [65]. Additionally, women may downplay their multifactorial needs and identities and strategically emphasize their victimization to obtain aid, undermining their ability to participate in nuanced discussions about GBV, SEA, and the other issues that affect them [66]. There are also general challenges to civil society engagement in conflict-affected settings, such as departure of civil society leaders and limited access to information or means of communication [67]. Inadequate legislative frameworks, elite capture (i.e. domination of the elite in participatory fora), bureaucratic and political cultures that do not value engagement by citizens or other affected populations, and government hostility to the affected group(s) can also undermine participation by women’s groups and broader civil society [67].

##### Local dispute resolution

Affected populations may also create their own systems to advance justice, such as local dispute resolution systems (DRS). Local DRS are often the only form of justice that is accessible; these are typically – but not always - informed by customary law brought from the country of origin or specific to particular sub-populations [32, 68]. They are generally not codified, and focus on compensation and conciliation [32, 69]. Such locally derived systems may be preferred by affected populations because they are more accessible and timely, have a high degree of cultural legitimacy, are familiar and understandable, and maintain social cohesion for residents who must continue to live in close quarters [32, 69].

However, the appropriateness of DRS in humanitarian settings, particularly for SRH and RR issues, is disputed. For example, one system converted cases of rape into questions of elopement, adultery, or dowry, sometimes resulting in raped women being imprisoned for having committed a supposed crime [32]. Many authors pointed out that customary dispute resolution systems can reflect and perpetuate existing power structures and may be biased against disadvantaged groups, particularly women. Moreover, they rarely adhere to international legal standards [32, 36, 68, 69].

Longer ethnographic studies show that these local systems of justice are not necessarily passively accepted by all residents; different factions may dispute authority with the balance of power changing regularly [56, 57]. Moreover, the jurisdiction of customary dispute resolution systems is limited to disputes between camp residents, and does not apply to disputes with external parties such as non-refugees, aid workers, and state officials, many of whom provide SRH services [32, 70]. In addition to the DRS, many camps also have camp resident committees and camp bylaws and regulations, yet we found little written on these and how they interacted with other systems of justice. Despite these shortcomings, many researchers and policymakers stated that given their prevalence and cultural relevance, customary justice can be key to improving access to justice in some contexts, depending on the issue being adjudicated and the customary justice system in question [68].

### Challenges relating to accountability for SRH and RR in humanitarian settings

Almost all papers at least referred to – if not explicitly focused on - challenges to realizing accountability in humanitarian contexts. Researchers, policy analysts, and practitioners point out that while there are multiple frameworks for accountability, there are myriad challenges to accountability in humanitarian assistance. Focus on indicators of accountability can contribute to box checking that disregards a longer-term vision of human rights, or that undermines the flexibility needed to respond to emergent challenges [6]. Some of the potential pitfalls of accountability are intrinsic to the humanitarian endeavour, as opposed to development contexts. Importantly, development aid typically aims to boost the capacity of the state and supports achievement of national priorities, while humanitarian aid may explicitly avoid the state and be based entirely on externally generated priorities [20, 98].

Other commonly invoked challenges relating to the inherent nature of humanitarian settings include lack of time for consultation in sudden onset disasters; the legal immunity of some workers; lack of flexibility in funding and programming, due in part to donor focus on speed and scale; prioritization of accountability to donors rather than to affected populations; poor understanding of power dynamics within affected populations, and difficulties learning these in a short time frame; the stated humanitarian commitment to neutrality; the unusual degree of information-sharing that would be required to mount effective inter-agency complaint mechanisms; tension between organizational autonomy and coordination; the “success cartel” and concomitant challenges to program-based learning and improvement; potential tension between security and transparency; lack of relevant skills and commitment among humanitarian providers; the fact that some of the most robust mechanisms, such as HAP, are voluntary; organizational inertia; and health and justice system weaknesses [21, 41, 49, 50, 52, 64, 98–100]. Monitoring and measurement – key tools for accountability – can be especially challenging in humanitarian contexts. For example, one paper assessed to what extent global level reproductive, maternal, newborn, and child health indicators are being met for Syrian IDPs and refugees in Jordan, Lebanon, Turkey and Syria [101]. The researchers were able to gather some data on some populations, but were stymied by lack of data about denominators given the dynamic setting, inconsistencies in definitions used, lack of data sharing, and lack of data collection [101].

Some of these challenges can be addressed, such as lack of relevant skills among humanitarian providers; others are inherent to the early emergency phase but may be addressed as the acute crisis situation stabilizes; and some are a matter of contestation within humanitarianism. As one example of a contested issue in humanitarianism, some humanitarian workers state that engagement of affected populations is transformative, while others argue that in reality, such engagement merely masks the perpetuation of power imbalances intrinsic to the humanitarian endeavour [50].

In addition to these general challenges to accountability in humanitarian settings, there are further challenges that are specific to SRH and RR. We reviewed over 70 papers that address SRH and RR in humanitarian settings (see Additional file 4), that, while not specific to accountability, included at least some explicit or implicit discussion of accountability. Many papers pointed to the legal and political environment as primary obstacles, such as lack of supportive SRH and GBV policies, the need for more government commitment, lack of availability of abortion, and governance challenges related to the fragmentation of the health care system. More generally, while they used different terms and frameworks, many of the papers discussed social norms in some way, including how health and social services may perpetuate harmful hierarchies related to gender, age, nationality, social class, ability, and ethnicity, resulting in disrespectful care, lower quality clinical care, and lower demand for care. Agency commitment to changing these norms may be undermined where SRH and RR are not domestic or institutional priorities but a donor condition [6, 93].

## Discussion

In the last 20 years, there has been moral reckoning, standards and guideline development, and program experiences related to accountability in humanitarian settings, including for SRH. The field is diverse and complex, as informal systems of accountability can reflect and perpetuate harmful norms; there is political and social contestation about accountability in humanitarian work and about SRH services; and the capacity and mandate of the host state and duration of crises varies considerably. Despite this complexity, international human rights law, professional principles, and minimum standards provide some universal architecture for accountability policy-making; and for program design, implementation, monitoring, and improvement. However, as our findings illustrate, institutional commitment to implementation, robust mechanisms to ensure implementation, and sanctions and access to remedy in the case of failure remain major gaps.

We did identify promising accountability approaches – some specific to SRH and some not - such as open and close-ended feedback from affected populations and practical application of standards for performance accountability. While significant effort went into developing and revising the MISP, we found few examples of programs that replicate or scale strategies to operationalize the MISP that showed some success, such as scorecards or quality improvement, including participatory quality improvement. Despite the fact that we searched grey literature, it is likely that there is significant institutional knowledge about MISP operationalization within humanitarian agencies that is not published in the peer-reviewed literature; our interviews suggested this to be the case, as many interviewees told us about how their organizations support MISP implementation. The peer-reviewed and grey literature papers suggested that institutional commitment and capacity at all levels are important enablers of accountability, but commitment and capacity may be hard to foster given staff turnover and the short termism associated with humanitarian work. In other words, the characteristics of most humanitarian situations make standard legal and performance accountability approaches more difficult.

The picture regarding access to justice is predictably muddy. Social, structural, and other impediments are significant, and vulnerability among survivors and other affected individuals is acute. The embedded legal, resource, and social obstacles to justice emerged repeatedly in reviewed articles and interviews, though several described programs that successfully chipped away at these impediments. The role of customary justice, given its prevalence and salience and potential to perpetuate harmful hierarchy, needs to be critically assessed for its potential to improve access to justice.

Some researchers and policy advocates state that the assumption that any humanitarian situation is a “blank slate” in which humanitarian actors provide seemingly neutral technocratic services can reproduce hierarchies based on gender, ethnicity, ability, and region of origin, among others [102]. The salience of local social dynamics was highlighted in relation to collecting evidence [38]; increasing awareness and reporting of GBV [79–81], training to improve the quality of services [48, 91], and the provision of safe abortion and SRH services [21, 103]. Some advocates believe that humanitarian actors should try to disrupt power dynamics to ensure access to services and protection for the most vulnerable [21, 98, 104]. Engaging with local civil society, particularly women’s groups, is offered as one way to affect power dynamics – thus ensuring accountability to all affected populations - and to make sure that services are culturally and otherwise relevant. However, some interviewees expressed concerns about humanitarian efforts to create or induce women’s groups, which can reportedly put members at risk, as the women face censure for their participation and consequent violation of social norms.

A diverse array of papers referred to accountability for SRH driven by affected populations themselves, but no paper provided a detailed overview of institutionalized, funded efforts to engage local civil society or to foster community driven solutions. Rather, most papers reviewed focused on interventions generated by the humanitarian sector itself, though a few of these had significant participatory elements. Reflecting this largely top down orientation, papers concentrate on accountability mechanisms within humanitarian work – whether on how services are provided, soliciting community feedback, around professional conduct, or how prosecutorial evidence is collected, with much less focus on supporting affected populations to develop critical consciousness about their position, understand their entitlements, or access justice.

The emphasis on tools or mechanisms for accountability with less attention to norms and change is analogous to discussions about accountability in development, where researchers point to disproportionate focus on ‘widgets’ rather than sustainably changing the norms, values, and patterns of interactions among the actors involved [105]. A few authors invoked a “beyond tools” approach to creating a culture of accountability [6, 21, 64]. The notion of a ‘culture of accountability’ is integrated to varying degrees in burgeoning guidance; for example, the discussions feeding into the 2016 World Humanitarian Summit addressed collective accountability and developing cultures of accountability within aid agencies [6].

### Limitations

Even though we included grey literature and interviews, we think it likely that the gap between what we were able to learn and on the ground reality is significant and points to the limitations of evidence reviews regarding the subject at hand. In other words, our analysis is limited by the fact that it is quite likely that there are strategies, successes, and challenges experienced by affected populations, humanitarian workers and agencies that we do not know about. Given that we comprehensively reviewed the peer-reviewed literature, the best way to overcome this limitation would likely be to interview additional stakeholders (we did not have that many stakeholders representing each category of respondent or area of expertise), or to conduct original empirical research. Moreover, comparatively fewer papers and resources focus on non-camp populations, such that we learned less about IDPs not in camps (including urban refugees) unrecognized refugees, migrants, and other groups ‘on the move’ who may face similar challenges to accountability for SRH and RR.

Moreover, there are limitations inherent to a scoping review. Our intent was to describe a heterogeneous body of literature from different disciplines on a topic, rather than to meticulously summarize primary research, so we did not assess the quality of the papers included. While this approach is acceptable (and some say required) for a scoping review, it introduces the possibility that our results were influenced by poor quality studies [106]. Because the scoping review process is more open and iterative than a systematic review, it is more vulnerable to the introduction of bias. While we were very careful about drawing conclusions from the literature reviewed, we could have unknowingly introduced bias by neglecting to follow up particular lines of enquiry. For example, our search terms may have excluded an important area of work. Our use of Google for the grey literature search means that we relied on Google’s algorithm to determine what was pertinent.

On the other hand, the scoping review methodology we followed allowed us to synthesize a diverse set of literature. This approach offers expertise and lessons from outside the public health field – such as development studies and law – to a public health discussion. The use of a wide variety of literature also surfaced issues, such as stigma, that are specific to SRH, as well challenges that are generalized to accountability in humanitarian settings, like short-termism or challenges in promoting and supporting local civil society engagement. Moreover, the grey literature and interviewees discussed emerging challenges, priorities, and program approaches that had seemingly not yet been addressed in a peer-reviewed article, or that are not typically the subject of global health research, such as feedback mechanisms in humanitarian settings.

### Next steps

Greater research and/or documentation is required in a few key areas, including the impact of both open ended and close ended community feedback mechanisms, the impact of customary justice in diverse customary justice systems, ways that accountability can be institutionalized and mainstreamed, the successes and challenges of efforts to provide stigmatized SRH services, and how to strike a balance between inducing community feedback and participation and not exposing vulnerable populations to social harms for violating social norms.

Commitment to localization within the humanitarian community provides opportunities for more consistent, sustained engagement of existing women’s groups and civil society more broadly. Donors and INGOs can better support local civil society to address basic humanitarian needs and to promote human rights accountability, such as by creating mechanisms to foster the engagement of these groups throughout the humanitarian response, creating funding streams for social accountability and programs to promote the voice of affected populations, and ensuring that funding opportunities are accessible to local NGOs, including those focused on women and girls [59, 107]. Moreover, while legal instruments, humanitarian principles, and codes of conduct often refer to the needs of especially marginalized groups, such as girls and adolescents, people with disabilities, and other minority groups among affected populations, we found few examples of accountability efforts that successfully addressed these issues; interviewees with expertise in this area confirmed that this remains an important gap.

In addition to further research or documentation, there should be greater cross-germination between the burgeoning community of people working on accountability for health in the development community and the smaller group of people addressing this issue in the humanitarian community. This cross-context exchange could include researchers, policy-makers, and advocates working at country and global levels. Given the increasing duration of displacement, the increase in climate related disasters, and the number of fragile states, the distinction between humanitarian and development can be ambiguous. Exchange may be particularly fruitful as many contexts straddle or vacillate between humanitarian and development.

## Conclusion

The importance of context is now a truism in development discourse. Explicating the context in terms of gender or other hierarchies that are often at the heart of accountability failures in SRH may be inherently more difficult in humanitarian settings, as the relationship between affected populations and duty bearers may be characterized by mistrust or just ignorance of the other. However, given the increasing prevalence of protracted crises, accountability deserves renewed attention, not only as a strategy to improve services in the present, but as a wedge for trust building and empowerment in transitions from humanitarian to development contexts. As a fundamental health need and determinant of gender equity, SRH and RR can be front and centre in these discussions.

## Supplementary information

**Additional file 1: Online Annex A.** List of codes.

**Additional file 2: **Online Annex B: Accountability for SRH in humanitarian settings

**Additional file 3:** Online Annex C: SRH in AAP documents

**Additional file 4:** Challenges identified in broader SRH in humanitarian literature

## Data Availability

All data generated or analysed during this study are included in this published article [and its supplementary information files].

## Abbreviations

AAP: Accountability to Affected Populations
BPHS: Basic Package of Health Services
CHS: Core Humanitarian Standard
DRS: Dispute Resolution Systems
GBV: Gender-Based Violence
HAP: Humanitarian Accountability Partnership
IASC: Inter-Agency Standing Committee
IAWG: Inter-Agency Working Group on Reproductive Health in Crises
IDP: Internally Displaced Person
MCH: Maternal and Child Health
MISP: Minimum Initial Service Package
PSEA: Protection against Sexual Exploitation and Abuse
RR: Reproductive Rights
SEA: Sexual Exploitation and Abuse
SRH: Sexual and Reproductive Health
