# Supplementary material for: Accountability strategies for sexual and reproductive health and reproductive rights in humanitarian settings: a scoping review
Source: Confl Health. 2020 Apr 7;14:18. doi: 10.1186/s13031-020-00264-2 (PMC7137319; doi:10.1186/s13031-020-00264-2)
Supplement: Supplementary file 1 — Additional file 1: Online Annex A. List of codes. [file 13031_2020_264_MOESM1_ESM.docx]

**Online Annex A: List of codes**

- - General barriers to accountability in humanitarian settings
  - Sexual exploitation and abuse
  - Access to justice
  - Service quality and quality improvement (includes patient satisfaction and patient defined quality)
  - Activism within affected populations
  - Engagement of local civil society/Women’s groups
  - Changing nature of humanitarian work (cash, remote management/localization, ICT)
  - Approach to gender (gender transformation etc.)
  - Customary/restorative justice
  - Discourse/media (focusing on narratives related to abortion, GBV, etc. and the way they shape accountability for SRHR or accountability more broadly in humanitarian)
  - Humanitarian agency mechanisms for responsiveness
  - Humanitarian feedback mechanisms
  - History/evolution of accountability to affected populations
  - Humanitarian/development nexus
  - Community based complaint mechanism
  - Facilitators of accountability
  - Affected populations’ awareness of rights/violations/entitlements
  - MISP
  - Perverse effects of accountability efforts
