# Supplementary material for: Accountability strategies for sexual and reproductive health and reproductive rights in humanitarian settings: a scoping review
Source: Confl Health. 2020 Apr 7;14:18. doi: 10.1186/s13031-020-00264-2 (PMC7137319; doi:10.1186/s13031-020-00264-2)
Supplement: Supplementary file 4 — Additional file 4: Challenges identified in broader SRH in humanitarian literature [file 13031_2020_264_MOESM4_ESM.pdf]

Title	Authors	Description	Definition of accountability	Is the framework voluntary?	To whom does it apply?	Does it have a formal relationship to other frameworks?	Relevant SRHR standards	Relevant gender standards	Mechanisms for reporting/ enforcement/ quality control
**ICRC Humanitarian Values and Response to Crisis (1995)**		This document attempts to set standards for behavior of organizations in humanitarian action. It underlines the importance of centering the work of humanitarian actors on the people in need, viewing them as partners to be consulted and instructed by, not as victims. Additional importance is placed on building upon local capacity when responding to a disaster and supporting work being done by national actors.		Yes	ICRC personnel; Open to all humanitarian organizations	No			Self-policing; offers a framework against which agencies can measure themselves.
**People in Aid "Code of Good Practice in the Management and Support of Aid Personnel" (2005)**		The Code is an important part of People In Aid's contribution to improving human resource management in the relief and development sector. The Code addresses the people-related components of risk management strategy, and is a quality tool which sits alongside others as agencies aim to improve their accountability to various key stakeholders, in People In Aid's case primarily to staff and volunteers. The Code offers a framework which will help assess, and if necessary improve, performance in human resource management.	NGOs openly and often acknowledge their accountability to two main stakeholders: donors and beneficiaries. People In Aid maintains that to truly satisfy their accountability to these two groups they must also be accountable to those who deliver their missions: their staff and volunteers.	Yes	It applies to agencies employing any combination of international staff (those working outside their own country), host country staff or volunteers.This revised Code will benefit every agency involved in humanitarian relief, development assistance or advocacy.	No			The verification process – a "social audit" In the open process which gave rise to the Code it was widely agreed by participating agencies that a verification process should be introduced to the Code to distinguish it from other Codes to which NGOs could sign up. It was decided that this would be in he form of a social audit, since the values at its heart (participation, accountability and transparency) are closest to the values of our sector.<br>A major objective of social audit is to work with selected stakeholders on the concerns they raise. The major stakeholder in the People In Aid Code process is staff. Staff are consulted during the process to ensure<br>that decision-makers and policy-writers both in HR and operational departments have data and opinion on<br>which to base their work.
**ECHA/ECPS UN and NGO Task Force on PSEA's SEA Victim Assistance Guide (2009)**		In order to implement the December 2007 UN Resolution mandating the assistance of the UN to victims of SEA by UN staff and related personnel, the UN and NGOs collaborated through an inter-agency task force to formulate a joint approach to victim assistance. This document describes that approach and provides instruction to country leadership to implement a SEA Victim Assistance Mechanism (SEA/VAM).	None provided	Yes	It is a guide for country leadership to design a system to assist and support survivors of SEA by UN/NGO/IGO personnel	Yes - it is meant to help operationalize the 2006 UN Statement of Commitment on Eliminating SEA, the 2007 resolution to assist victims of SEA, and the IASC Six Core Principles relating to SEA	Maintain a survivor-centered approach.<br>Provide assistance to all persons victimized by staff or related personnel of UN/NGOs/IGOs.<br>Assistance and support is to be offered based on individual needs directly arising from SEA.<br>Children born as a result of SEA should be entitled to receive medical, legal and psychosocial care.		Ideally works in tandem with a PSEA network.<br>Victim Support Facilitators (similar to case workers) will help guide complainants, victims, or children born from SEA through access to services.<br>Entry into the SEA/VAM should be offered to all victims of SEA.<br>SEA/VAM should be separate from the allegation and investigation processes.
**HAP Accountability & Quality Management (2010)**		The HAP Standard helps organisations that assist or act on behalf of people affected by or prone to disasters, conflict, poverty or other crises to design, implement, assess, improve and recognise accountable programmes. It describes how to establish a commitment to accountability and the processes that will deliver quality programmes for the people who experience them first hand.	Accountability: the means through which power is used responsibly. It is a process of taking account of, and being held accountable by, different stakeholders, and primarily those who are affected by the exercise of power.	Yes	The HAP Standard can apply to all types of local, national and international organisations that assist or act on behalf of people affected by or prone to disasters, conflict, poverty or other crises, including HAP members and non-members, multi-mandate organisations, and organisations that deliver direct assistance and those that work with partners.	The HAP Standard is intended to complement other standards that relate to accountability to people affected by different types of crises, as well as issue-specific standards and guidelines on quality management systems, human rights, and protection from abuse and corruption.<br><br>In particular, the HAP Standard is intended to complement the work by People In Aid, the Sphere Project, the Emergency Capacity Building Project, Groupe URD (Urgence, Réhabilitation, Développement), the Active Learning Network for Accountability and Performance in Humanitarian Action (ALNAP) and national self-regulatory schemes that focus on accountability and quality in the non-profit sector.			• Establishing and delivering on commitments<br><br>• Staff competency:The organisation ensures that staff have competencies that enable them to meet the organisation's commitments.<br><br>• Sharing information:The organisation ensures that the people it aims to assist and other stakeholders have access to timely, relevant and clear information about the organisation and its activities.<br><br>• Participation:The organisation listens to the people it aims to assist, incorporating their views and analysis in programme decisions.<br><br>• Handling complaints: The organisation enables the people it aims to assist and other stakeholders to raise complaints and receive a response through an effective, accessible and safe process.<br><br>• Learning and continual improvement: The organisation learns from experience to continually improve its performance.
**IASC 2011Commtment to Accountability to Affected Populations (2011)**		Ithe 2011 ASC Principals meeting endorsed five Commitments to Accountability to Affected Populations (CAAP), and agreed to incorporate them into the policies and operational guidelines of their organizations and promote them with operational partners, within Humanitarian Country Teams and amongst cluster members.	The document lays out five components to accountability: 1) Leadership/Governance, 2) Transparency, 3) Feedback and Complaints, 4) Participation, 5) Design, Monitoring & Evaluation	Yes	IASC Principal agencies and all those who opt in				Actively seek the views of affected populations to improve policy and practice in programming, ensuring that feedback and complaints mechanisms are streamlined, appropriate and robust enough to deal with (communicate, receive, process, respond to and learn from) complaints about breaches in policy and stakeholder dissatisfaction.<br><br>Design, monitor and evaluate the goals and objectives of programmes with the involvement of affected populations, feeding learning back into the organisation on an ongoing basis and reporting on the results of the process.
**Granada Report (2011)**		In September 2009, the WHO, UNFPA and the Andalusian School of Public Health convened a global consultation in Granada, Spain to address the gap in SRH service provision during protracted crisis and recovery. This meeting produced the "Granada Consensus," a statement that highlights four prioritiy areas to be addressed in order to facilitate the sustainable provision of SRH services in protracted crises. The WHO and UNFPA then organized a follow-up meeting of 20 experts to promote the implementation of the Granada Consensus. This publication presents the work undertaken during these two gatherings as well as the conclusions, outcomes and recommendations that were produced.		Yes	Provides guidance for humanitarian agencies and governments	No	Calls for mainstreaming of SRH in all health policies during the recovery period and/or a protracted crisis; for consolidation and expansion of SRH services in protracted crises & recovery (full implementation of the MISP as a starting point); for commitment from humanitarian & development actors to bridge service delivery and funding gaps between humanitarian and recovery phases. "Recognize and support the leadership role of national and local authorities, communities and beneficiaries in ensuring sexual and reproductive health."		

**Tools to assist in implementing the IASC AAP commitments (2012)**	The analysis and planning tool represents a selective synthesis of key industry standards and frameworks, namely the HAP Standard, the Sphere Core Standards, the People In Aid Code, the Global Humanitarian Platform's Principles of Partnership5 and CDA's Do No Harm Framework with the draft IASC operational framework and accountability commitments to form a "meta-framework" for understanding in greater depth what each of the commitments should mean in practice. It highlights some of the basic and globally agreed upon fundamentals of quality and accountability in humanitarian service delivery. This tool is not in itself an industry standard, however, it draws upon the core and common themes and issues across the standards that apply directly or indirectly to the concept of accountability to affected populations and borrows heavily from the indicators and requirements therein to guide measurement of performance and current status.	The tool organises analysis under the five key pillars of the IASC commitments, and adds one additional crosscutting area; that of working with partners and other stakeholders. This section incorporates some considerations highlighted in the source material when considering how to best ensure accountability and quality commitments reach affected communities given humanitarian organisations work as a part of a wider system and often deliver	Yes	Humanitarian agencies	It is a sythesis of: the HAP Standard, the Sphere Core Standards, the People In Aid Code, the Global Humanitarian Platform's Principles of Partnership and CDA's Do No Harm Framework with the draft IASC operational framework and accountability commitments	A code of conduct exists that explicitly addresses protection of people the agency seeks to assist from sexual abuse, corruption, exploitation and other human rights violations, and:<br>· The code specifically refers to staff, volunteers and partners not exploiting and abusing people, and the responsibility of staff to report abuses<br>· The codes are shared with affected communities so that they know what to hold workers, partner staff and volunteers to account for<br>· Safe and effective grievance procedures exist and the agency takes appropriate disciplinary action against workers following confirmed violation of the code of conduct		Self-Assessment Against the CAAP ranking tool:The self-assessment tool breaks down each of the indicators within the analysis and planning tool to provide practical assistance for any user group to understand and measure them. The self-assessment tool has been developed to further break down each of the indicators within the analysis and planning tool and is structured to assist in highlighting priority areas for action and areas for potential "quick wins".<br><br>Rapid Cluster Accountability Review tool - This brief tool provides some key and focused areas for assessment from an HCT, cluster, or interagency perspective. Reviewing AAP from this vantage point will be more likely to highlight, for example, issues of coordination, the potential for agencies stronger in AAP to lead and assist those yet to incorporate it into their practice, and areas most suited for collaboration and a consistent approach.
**IASC AAP Operational Framework (2013)**	The Operational Framework summarizes the key concepts for making programming at the field level more accountable to affected populations. The framework is designed to assist implementing agencies both individually and in groups to find practical entry points for improving accountability to affected populations.		Yes	Humanitarian agencies	Yes - IASC 2011 Commitment to AAP. Also to be used in conjunction with the IASC Accountability Commitment Analysis Tool, the HAP 2010 Standard in Accountability and Quality Management, the Minimum Operating Standards for Protection from Sexual Exploitation and Abuse, or locally developed and tailored tools.			•Mainstreaming accountability commitments into recruitment and training<br>•Amend partnership agreements to include accountability<br>•Systematically communicate with affected populations using relevant feedback and communication mechanisms<br>•Develop if necessary and/or support multi-agency response communications initiatives. Implement communications projects that already deliver on a response-wide level, (including IFRC's SMS system, the radio models developed by IFRC and the BBC WST, support to local media and other actors).<br>Affected populations have opportunity to register complaints, provide feedback and to get a response<br>•Include accountability to affected populations in job descriptions, staff development and appraisal mechanisms, and particularly in the Terms of Reference of the Cluster Coordinators<br>•Ensure that accountability to affected populations is effectively integrated within needs assessment methodology, including joint needs assessments<br>•Design and implement feedback mechanisms in consultation with local communities and inform all stakeholders how they function<br>•Ensure that accountability to affected populations is effectively integrated within systems for project design and planning<br>•Ensure that accountability to affected populations is effectively integrated throughout the implementation of projects
**Community of Practice on Community Engagement (2015)**	The CoP is expected to provide strategic advice and technical support to the existing field level TWGs including improving two-way communication platforms, feedback avenues and closing the communication loop mechanisms.		Yes	All those working on community engagement such as UN agencies, international and local NGOs, private sector (specifically the telecommunication companies and online media networks), national and local government agency like the PIA, CSOs, faith-based groups, academe and the traditional media including mainstream and the humanitarian press				Set of guidelines that are divided between actions to be taken in an emergency vs non-emergency/preparedness setting<br><br>CoP should work with the Humanitarian Communications Group (HCG) for wide coverage and delivery of the key messages, and expand channels for feedback to ensure voices of the affected communities are integrated into public information, response-wide advocacy materials and action points from the HCT<br><br>Members should agree and identify local champions on community engagement in various agencies to help mainstream and replicate good practices on communication, accountability and community participation for future emergency response.<br><br>Members should support a joint/coordinated training on basic communication, accountability and community participation activities for agencies or clusters as part of capacity building.<br><br>Members should source and share existing good initiatives, best practices and research undertakings on communications, accountability and community participation (like learning reviews and case studies<br><br>Members should identify and prioritize support mechanisms in which humanitarian responders and field level TWG can listen and provide the necessary action points to the needs and concerns of the affected communities especially on access to information, reliable feedback mechanism and trusted channel for participation.
**IASC Directors Group Protection & Accountability (2015)**	This note sets out actions to be undertaken throughout the humanitarian programme cycle (HPC) to fulfill commitments on Accountability to Affected Populations (AAP) and to ensure that protection is central to humanitarian response. It sets out the fundamental link between accountability systems and protection in humanitarian action, and then describes, for each stage of the HPC, the accountability mechanisms that should be established and actions required at country-level, as the foundation for appropriate and effective programming to achieve improved protection outcomes for crisis-affected communities.	Accountability refers to the responsible use of power, combined with effective and quality programming that recognizes the community's dignity, capacity, and ability for self-determination.	Yes	Humanitarian Coordinators and Humanitarian Country Team members (including in their role as cluster lead agencies)	Yes - 2011 AAP			Key protection risks and needs are identified and analysed through the participation of a broad and representative spectrum of the community in a manner that ensures meaningful participation of all age, gender and diversity groups.<br><br>Priorities and desired protection outcomes are identified, pursued and achieved collectively across sectors/clusters and with communities through meaningful participation, which reinforces a rights-based approach, empowers communities, recognizes differences in age, gender and diversity, guarantees transparent responses to community feedback and leverages the complementary roles, expertise and mandates of humanitarian actors.<br><br>Programme implementation and monitoring is done in participation with communities in a manner that enables their meaningful feedback and contribution, while at the same time, facilitates an ongoing analysis of protection risks that translates into action and informs adjustments to the response and future strategic planning.

**Core Humanitarian Standard on Quality and Accountability (2015)**	The Core Humanitarian Standard on Quality and Accountability (CHS) sets out Nine Commitments that organisations and individuals involved in humanitarian response can use to improve the quality and effectiveness of the assistance they provide. It also facilitates greater accountability to communities and people affected by crisis: knowing what humanitarian organisations have committed to will enable them to hold those organisations to account.	The process of using power responsibly, taking account of, and being held accountable by, different stakeholders, and primarily those who are affected by the exercise of such power.		The CHS can be promoted and implemented by individuals, organisations, coordinating bodies, consortia and other groups undertaking or contributing to humanitarian action. Although primarily intended for the humanitarian sector, the CHS can be used by any organisation to bring better quality and greater accountability to all aspects of its work with communities and people affected by crisis.	The CHS is the result of a global consultation process. It draws together key elements of existing humanitarian standards and commitments. These include but are not limited to:<br>• The Code of Conduct for The International Red Cross and Red Crescent Movement and NGOs in Disaster Relief;<br>• The 2010 HAP Standard in Accountability and Quality Management;<br>• The People In Aid Code of Good Practice in the Management and Support of Aid Personnel;<br>• The Sphere Handbook Core Standards and the Humanitarian Charter;<br>• The Quality COMPAS;<br>• The Inter-Agency Standing Committee Commitments on Accountability to Affected People/Populations (CAAPs); and<br>• The Organisation for Economic Co-operation and Development's (OECD) Development Assistance Committee (DAC) Criteria for Evaluating Development and Humanitarian Assistance.	Identify and act upon potential or actual unintended negative effects in a timely and systematic manner, including in the areas including sexual exploitation and abuse by staff; The complaints-handling process for communities and people affected by crisis is documented and in place. The process should cover programming, sexual exploitation and abuse, and other abuses of power.	Encourage and facilitate communities and people affected by crisis to provide feedback on their level of satisfaction with the quality and effectiveness of the assistance received, paying particular attention to the gender, age and diversity of those giving feedback.	Key Actions<br>1.1 Conduct a systematic, objective and ongoing analysis of the context and stakeholders.<br>1.2 Design and implement appropriate programmes based on an impartial<br>assessment of needs and risks, and an understanding of the vulnerabilities and<br>capacities of different groups<br>1.3 Adapt programmes to changing needs, capacities and context.<br>Organisational Responsibilities<br>1.4 Policies commit to providing impartial assistance based on the needs and<br>capacities of communities and people affected by crisis.<br>1.5 Policies set out commitments which take into account the diversity of communities,<br>including disadvantaged or marginalised people, and to collect disaggregated data.<br>1.6 Processes are in place to ensure an appropriate ongoing analysis of the context.
**World Humanitarian Summit (2016)**	On 23 and 24 May 2016, the first World Humanitarian Summit was convened in Istanbul, Turkey: the first time in the history of the United Nations that representatives of Member States, non-governmental organizations, civil society, populations affected by crises, the private sector and international organizations came together on such a significant scale.<br><br>Some key themes that emerged included: protecting civilians & minimizing human suffering; enhancing respect for international human rights & refugee law; reducing forced displacement; women and girls as agents of change; inclusion; "A new way of working" - national & local leadership & ownership (including improved accountability to people affected by crises); transcend the humanitarian-development divide; risk & data-driving planning, programming & decision-making; strengthening the resource base & increasing efficiency; and documenting/reproting on commitments made at summit			UN Member States		New financing and programmes were pledged for women's and girls' education, training, livelihood support and access to quality, comprehensive sexual and reproductive health care. Governments committed themselves to undertake legislative reforms to ensure greater protection for women of all ages and girls from all forms of gender-based violence, no matter the setting.	The summit urged all stakeholders, in the implementation of their commitments, to ensure that they promote gender equality and uphold women's and girls' rights and that they develop and make publically available specific plans and concrete and time-bound targets to implement them.	An online Platform for Action, Commitments and Transformation will be established to enable ongoing engagement and to offer guidance on making new commitments. The Platform will also serve as the main sources of information to analyse and report on progress, and will also:<br>(a) Serve as a transparency tool by publicly displaying all individual and collective commitments, alignments with core commitments and initiatives related to the Agenda for Humanity;<br>(b) Be based on self-reporting, and will track continued progress by enabling any stakeholders to observe commitments and progress made by others: it will be interactive, allowing users to search commitments and analyse trends;
**The Grand Bargain (2016)**	The Grand Bargain is an agreement between more than 30 of the biggest donors and aid providers, which aims to get more means into the hands of people in need. The Grand Bargain includes a series of changes in the working practices of donors and aid organisations that would deliver an extra billion dollars over five years for people in need of humanitarian aid. The Grand Bargain commits donors and aid organizations to providing 25 per cent of global humanitarian funding to local and national responders by 2020, along with more un-earmarked money, and increased multi-year funding to ensure greater predictability and continuity in humanitarian response, among other commitments. It also includes commitments to increase inclusion of beneficiaries in planning and decision-making.		Yes	Humanitarian agencies and donors	Originated in "Too important to fail: addressing the humanitarian financing gap"; Helps to promote the Core Humanitarian Standard and the IASC Commitments to Accountability to Affected Populations.			Use of International Aid Transparency Initiative (IATI) for data sharing, transparency, and limiting duplication between agencies; harmonizing and simplifying reporting requirements across agencies and donors
**Revised Commitments on Accountability to Affected Populations (2017)**	In 2011, the IASC principals agreed to five Commitments on Accountability to Affected Populations (CAAP) as part of a framework for engagement with communities. The revised version was developed and endorsed by the IASC Principals in 2017 to reflect essential developments such as the Core Humanitarian Standard(CHS), the work done by the IASC on Inter-Agency community based complaints mechanisms including PSEA and the importance of meaningful collaboration with local stakeholders, which came out as a priority recommendation from the 2016 World Humanitarian Summit and in the Grand Bargain.	The document lays out five components to accountability:<br>1) Leadership<br>2) Partnership & participation<br>3) Information, feedback & action<br>4) Results	Yes	IASC Principal agencies and all those who opt in	Yes -replaces IASC 2011 Commitment to AAP	Enforce, institutionalize, and integrate AAP approaches in the Humanitarian Program Cycle and strategic planning processes, including for PSEA	Adopt agency mechanisms that feed into and support collective/people-centered approaches that enable women, girls, boys, men, including the most marginalised and at-risk people among affected communtiies, to participate in and play an active role in decisions that will impact their lives	Measure AAP and PSEA results at the agency and collective level, including through standards such as the Core Humanitarian Standard, the Minimum Operating Standards on PSEA, and the Best Practice Guide to establish Inter-Agecny Community-Based Complaint Mechanisms<br><br>Adopt agency mechanisms that feed into and support collective and participatory approaches that address feedback and lead to corrective action.

**Cluster Operational Guidance on AAP (2017)**		This practical guidance is designed to assist Health Cluster Coordination Teams in leading, with cluster partners, emergency responses that have strong and robust accountability systems, through which affected populations can increasingly influence the type, delivery and quality of assistance they receive.<br>The guidance is organised according to the phases of the Humanitarian Programme Cycle (HPC), describing the relevant Core Humanitarian Standards (CHS), with proposed Health Cluster focussed activities under each phase, for how participation from affected populations can be improved. Standard indicators selected from the Inter-Agency Committee (IASC) AAP Toolkit1, are proposed to measure how well these activities are implemented. Each section provides an example of good practice, which can be used by Health Cluster Coordination Teams to lead in designing country specific AAP plans in the cluster.	•Taking account; giving people the opportunity to influence decisions which need to be made at different phases of the HPC, taking into account the diversity of the community and the views and opinions of the most vulnerable being equally weighed and considered. This means actors should go to thec ommunity to solicit opinions and thoughts from a broad spectrum of women, girls, boys and men of different age groups. It is not a passive exercise, where agencies might expect the population to give an opinion if they so desire. This assumes that everyone has the same freedom of expression which we know to be untrue.<br>• Giving account; refers to actors providing information to the community throughout the HPC and outlining what plans and commitments are and how and why decisions were made and what the process was. It includes what beneficiary selection<br>criteria are, how these were agreed, but also what programmatic objectives are and how these were set.<br>• Being held to account; allows affected people an opportunity to assess the quality of the response of an agency and how relevant activities have been, to assess how these activities have been implemented and to provide feedback on how well the activities have been addressing their needs.	Yes	Health cluster coordination teams	Yes - IASC 2011 Commitment to AAP		Consult with specialised agencies and available focal points to ensure AAP, gender, protection and diversity issues are appropriately addressed by Health Cluster, including prevention of sexual exploitation and abuse<br>(PSEA)<br><br>Establish a TWG to support the Health Cluster to implement a people centred approach –<br>mainstreaming AAP, gender, protection and diversity into the emergency health response.<br><br>Regularly review AAP, gender, protection and diversity issues in Health Cluster meetings<br><br>Consult with specialised agencies and available focal points and experts on appropriately incorporating AAP, gender, protection and diversity in the Health Cluster Preparedness Plan.<br><br>Conduct an analysis of AAP, gender, protection and diversity related findings – taking into account the differing health needs of women, men, girls, boys older people, persons with disabilities and other specific groups at risk of discrimination or who are particularly vulnerable.	Share other relevant Health Cluster information with affected communities – including conduct to be expected from Health Cluster partners (codes of conduct) as well as affected population rights and channels to provide feedback and complaints.<br><br>Communicate back to the community on how these community engagement and feedback / complaints mechanisms are working and ensure communities are informed on action that has been taken by the cluster in response to complaints and feedback from affected population(s).<br><br>Ensure that affected communities are informed through preferred communication channels, when changes are made to the health programme schedule – eg. when mobile clinic services postponed.<br><br>Identify trends in the Health Cluster community engagement mechanisms and feedback and complaints mechanisms and ensure these trends are informing strategic and programmatic decisions.<br><br>Performthe Health Cluster Performance Monitoring (CCPM) exercis5 with involvement of national NGOs and community consultation to review collective strengths and weaknesses against AAP,gender, protection<br>and diversity targets – to guide adjustments/ improvements.
**Sphere Handbook (2018 update)**		The Handbook reflects Sphere's commitment to a principled and rights-based humanitarian response. It is based on fundamental respect for people's right to be fully involved in decisions regarding their recovery.<br>The four foundation chapters outline the ethical, legal and practical basis for humanitarian response. They underpin all technical sectors and programmes. They describe commitments and processes to ensure a good quality humanitarian response, and encourage responders to be accountable to those affected by their actions. These chapters help the user apply the Minimum Standards more effectively in any context.	"8. We offer our services in the belief that the affected population is at the centre of humanitarian action, and recognise that their active participation is essential to providing assistance in ways that best meet their needs, including those of vulnerable and socially excluded people. We will endeavour to support local efforts to prevent, prepare for and respond to disaster and to the effects of conflict, and to reinforce the capacities of local actors at all levels. 12. For our part, we undertake to make our responses more effective, appropriate and accountable through sound assessment and monitoring of the evolving local context, through transparency of information and decision-making, and through more effective coordination and collaboration with other relevant actors at all levels, as detailed in the Core Humanitarian Standard and the Minimum Standards. In particular, we commit to working in partnership with affected populations, emphasising their active participation in the response. We acknowledge that our fundamental ccountability must be to those we seek to assist."	Yes	Humanitarian agencies	Yes: It builds on the 1994 Code of Conduct for the International Red Cross and Red Crescent Movement and Non-Governmental Organisations (NGOs) in<br>Disaster Relief. And the following are listed as complementary standards: •• Livestock Emergency Guidelines and Standards (LEGS): LEGS Project<br>•• Minimum Standards for Child Protection in Humanitarian Action (CPMS):<br>Alliance for Child Protection in Humanitarian Action<br>•• Minimum Standards for Education: Preparedness, Response, Recovery:<br>Inter-Agency Network for Education in Emergencies (INEE)<br>•• Minimum Economic Recovery Standards (MERS): Small Enterprise Education<br>and Promotion (SEEP) Network<br>•• Minimum Standard for Market Analysis (MISMA): Cash Learning<br>Partnership (CaLP)<br>•• Humanitarian Inclusion Standards for Older People and People with<br>Disabilities: Age and Disability Consortium	1.3: Menstrual hygiene management and incontinence: Women and girls of menstruating age, and males and females with incontinence, have access to hygiene products and WASH facilities that support their dignity and well-being.<br>2.3.1: Reproductive, maternal and newborn healthcare: People have access to healthcare and family planning that prevents excessive maternal and newborn morbidity and mortality.<br>2.3.2: Sexual violence and clinical management of rape: People have access to healthcare that is safe and responds to the needs of survivors of sexual violence.<br>2.3.3: HIV: People have access to healthcare that prevents transmission and reduces morbidity and mortality due to HIV.	Gender is called out throughout the handbook as a factor that needs to be considered when planning programs, delivering aid, hiring staff, etc.There is significant discussion of GBV. But no individual standards specifically aimed at gender.	Monitoring, evaluation, accountability and learning<br>Monitoring, evaluation, accountability and learning (MEAL) supports timely and evidence-based management decisions. It allows humanitarian programmes to adjust to changing contexts. All of the Minimum Standards have indicators that can be monitored to determine whether they are being achieved, whether they are being achieved equitably for all segments of a population, or how much more needs to be done. Evaluation supports learning to improve policy and future practice,<br>and promotes accountability. MEAL systems also contribute to broader learning efforts related to effective humanitarian action.<br><br>C**ore Commitment 4: Communities and people affected by crisis know their rights and entitlements, have access to information and participate in decisions that affect them.** (contains information about providing information to affected communities about the services provided, communicating in approporiate languages and formats, participation and involvement of communities in planning activites and soliciting feedback from communities)<br>**Core commitment 5: Communities and people affected by crisis have access to safe and responsive mechanisms to handle complaints** (contains information about the importance of maintaining a complaints mechanism, how to go about setting one up, how to respond to complaints, and addresses organizational culture & staff codes of conduct
**Time to Listen: Hearing People on the Receiving End of International Aid (2012)**	Mary B. Anderson, Dayna Brown and Isabella Jean	This book captures the experiences and voices of over 6,000 people who have received international assistance, observed the effects of aid efforts, or been involved in providing aid.	No specific definition has been provided.					The evidence can be summarized in two essential points. 1) Pople who live in recipient societies view the international delivery system as turning them to objects of others' decision-making and planning, rather than engaging them as subjects in their society's progress. 2) No universal blueprint exists for creating peace or promoting humanitarianism, good governence, or development. Standard policies and procedures and prepackaged programs the international assistance system offers do not adequately reflect differences in the contexts where aid is provided.	
**Closing the Loop: Effective Feedback in Humanitarian Contexts, Practitionar Guidance (2014)**	Francesca Bonino, Isabella Jean and Paul Knox Clarke	This guidance is based on the results of an action research project that looked at different agencies' experiences in setting up and using feedback mechanisms in operational humanitarian contexts. Evidence was gathered through desk research and field visits.	A feedback mechanism is seen as effective if, at minimum, it supports the collection, acknowledgement, analysis and response to the feedback received, thus forming a closed feedback loop.				Considering the way in which sensitive information like sexual abuse will be addressed in advance by developing a procedure, for instance by making sure that agency staff working on a protection, sexual exploitation and abuse (SEA) and gender mainstreaming portfolio are, at minimum, made aware of how the feedback mechanism works.	A mix of staff genders to facilitate feedback collection and response to communities	
**Rhetoric or Reality? Putting Affected People at the Centre of Humanitarian Action (2014)**	Dayna Brown and Antonio Donini	This paper summarises current understandings of methods of and approaches to engaging with crisis-affected people in humanitarian action.	This paper follow HAP definition of accountability as 'the means through which power is used responsibly. It is a process of taking into account the views of, and being held accountable by different stakeholders, and primarily the people affected by authority or power' (HAP, 2010: 1). This paper uses 'engagement' as a term to cover all instances of people in crisis-affected communities becoming involved in planning and implementing responses to the crises affecting them. This broad definition covers the entire range of intentional interactions between those providing humanitarian aid and affected people, including activities focused on communication, accountability and participation. This definition also covers actions taken by local actors and crisisaffected people themselves to respond to a crisis directly without the intervention of international humanitarian organisations.					Participation is considered to lead to increased gender and social equality. Some humanitarian agencies explicitely recoginise these potential benefits in policy and programming. For example, World Food Programme policy calls for the use of participatory approach to overcome gender inequalities by creating opportunities for both women's and men's voices to be heard.	

**On the Road to Istanbul: How Can the World Humanitarian Summit Make Humanitarian Response More Effective (2015)**	CHS Alliance	This report presents ideas and perspectives from initiatives such as the Core humanitarian Standard on Quality and Accountability (CHS) to enrich the World Humanitarian Summit (WHS) in a way that truly put people at the centre of humanitarian action. This report discusses the interaction between accountability and effectiveness from different perspectives, and provides suggestions on actions the sector can take to maintain and improve the relevance of its work.	Accountability is considered by WHS as a key driver of humanitarian effectivenss. The report reviwes a number of definitions of accoutability, used in different contexts. The CHS Alliance conception of accountability is viwed to touch on several drivers of effectiveness for crisis-affected communities, including access to information, meaningful participation, opportunities to complain and give feedback, to receive a response, programme adaptation, and continuous improvement. Being accountable is about taking responsibility for actions (and inaction), results, behaviours, successes, failures, mistakes, and for learning (not just gathering) lessons. Accountability does not flow only 'upwards' to donors or 'downwards' to communities, but rather in all directions between people and organisations who have a relationship to one another.	1) The extortion of sexual favors in return for aid is expressed as a type of power abuse, defined as non-financial corruption, which will not be reflected in financial accounts and other formal documentation. 2) Requesting sexual favors from local authorities to include people on a beneficiary list is considered a problem in targeting and selection of beneficiaries. 3) Setting up specific policies and guidelines to prevent and report sexual exploitation and abuse, covering all staff, volunteers and partners as well as beneficiaries and communities affected by crisis is considered as a tool to combat corruption. 4) Recruitment process failing to spot sexual predators is considered to decline motivation and performance and increase staff turnover.	1) Based on the principle of impartiality, humanitarian action must be carried out on the basis of need alone, making no distinction on the basis of gender among other characteristics (nationality, race, religious belief, class or political opinions). 2) Proximity to affected communities is a prerequisite and driver of effectiveness because it helps to assess people's material and protection needs based on tehir specific vulnerabilties due to their age, gender, disabilities, etc. 3) Among other factors (i.e. age, ethnicity, culture and geography), gender is also considered as an importnat factor to determine the the type, weight and quantity of goods provided to the affected people in emergicny. 4) There is an increasing requirement of many funding and partnership agreements to demonstrate that gender as well as age and ability are considered in programme design, which has led to greater awareness of the importnace of these issues - although wide-scale and consistent application of gender analysis in programming is still lacking. It is suggested that satisfaction type questions be promoted in policy instruments, standards and donor requirements and this data be disaggregated by age and gender.
**Leaving No One Behind: Humanitarian Effectiveness in the Age of the Sustainable Development Goals (2015)**	OCHA	This study highlights 12 of the elements that are critical to effective humanitarian assistance and protection, and describes five overarching shifts in mind-set and approach that can contribute to imprvoment in supporting people in crisis, as well as moving people out of crisis. The findings are based on a 1,600-person global survey, six country visits that included hundreds of interviews, and other consultations.	The study mentions a number of accountability mechanisms, such as the International Criminal Court, Security Council-mandated comissionons of inquiry, and national or ad hoc tribunals. The study proposes that a global accountability framework be formulated to track progress on improving specific aspects of humanitarian effectiveness, used to inform interagency and intergovernmental processes as well as operational and policy options in crises. As a contribution to this accountability framework, the study proposes a set of "guiding principles" that highlight the main changes in relation to the study's 12 elements of effectiveness.	1) Sexual violence and gender-based violence (SGBV) is considered as a critical concern affecting whole communities, with women and adolescent girls being disporportionately affected. 2) Humanitarian crises, both conflicts and natural disasters, is considered to exacerbate and intensify various forms of SGBV including trafficking, early marriages and domestic violence. 3) While humanitarian organisations hold a commitment on zero tolerance on sexual exploitation and abuse, its continued occurrence remains a major barrier to progress for protection and gender equality outcomes.	1) A gender analysis is recommended to identify the unique coping strategies and capacities of women, girls, boys, and men as a bsis for gender equality programming. 2) In order to enhance accountability and effectiveness in accessing and addressing the needs of women, men, girls and boys appropriately, it is recommended to include Gender Market throughout all phases of humanitarian action, inclding monitoring and evaluation.
**Mainstreaming of Accountability to Communities: An Operational Case Study (2017)**	Sarah Cechvala	This report docments Kenya Red Cross Society's (KRCS) experience mainstreaming of the Accountability to Communities (AtC) framework across the organization in order to highlight the successes and challenges experienced by KRCS and provide practical lessonns baount factors that may enable a successful mainstreaming process.	The Accountability to Communities (AtC) initiative provides a framework and a set of minimum standards or pillars, including transparency, participation, feedback mechanisms, and community-led monitoring and evaluation. Each pillar is designed to provide a common goal for all programs and is expected to be integrated across all program lifecycles and emergency operations.		When designing the feedback channels, the process should be inclusive and engage vulnerable populations, paying attention to gender, s-e and other power dynamics.
**Aid Exits and Locally-led Development (2018)**	USAID	This report is based on a research in which 95 participants from 40 countries engaged and responded to prompts organised around daily themes ond aid exits and locally-led development. This report reflects on that exchange, identifies consensus where it appeared, and shares key nuances and insights from individuals.	Accountability is considered both toward the sources of funds, whether government or private donors, as well as those served by aid actors.		
**Private Sector: Who is Accountable? (2018)**	IAP	This report address the following questions: Can the private sector be held accountable for protecting women's, children's and adolescents' health? And if so, who is responsible for holding them to account, and what are the mechanisms for doing so? The scope is narrowed to for-profit actors operating in the health sector and/or having significant impact on women's, children's and adolescents' health across the pillars of the Global Strategy - Survive, thrive and transform.	The IAP approach captures the full cycle of accountability—monitor, review, act and remedy—building on the approaches of the Commission on Information and Accountability for Women's and Children's Health, and of the Independent Expert Review Group. The IAP's Unified Accountability Framework for the Global Strategy shows how levels of monitoring and review are layered and intertwined, from the national to the global.	1) Most private sector commitments fall under the survive and thrive pillars of the Global Strategy. 2) Under the survive pillar, many focus on newborn mortality, followed by child mortality and maternal mortality. Only one company addresses adolescent mortality; another has pledged to combat cervical cancer. 3) Commitments under the thrive pillar show strong support for essential health services, particularly SRH and to combat malnutrition; others address quality of care. Some companies focus on women's SRH, through services for their employees, covering global supply chains and providing parental leave. One of the few commitments to adolescents is made in the form of low-priced menstrual pads for girls in Rwanda who might otherwise miss days in school. Another one is promoting adolescents' access to contraceptives as well as unbiased sexuality education as part of World Contraception Day. 4) All the companies supporting SRH services for their workers conduct satisfaction surveys. 5) Financial mechanisms can enhance accountability, but their effectiveness depends on how private sector service delivery is structured and regulated. 6) While health insurance is generally associated with improved use of maternal health services, its effects on quality of maternal health care and on outcomes for women and newborns is understudied. 7) Regarding the role of health insurance, there is also the problem of narrow packages that cherrypick among the services covered, especially in women's SRH. 8) To overcome the socio-cultural and gender barriers and facilitate access by women and adolescent girls to reproductive health commodities, some countries no longer require prescriptions for selected contraceptives. 9) While abuse during pregnancy is not uncommon, it is often ignored in maternal health care.	1) The transform pillar of the Global Strategy has received less attention, with only 19 commitments; ten of these address gender equality, with only one on combatting violence against women. 2) 49 countries lack legislation to against physical and/or sexual violence by an intimate partner and 59 countries lack legislation against sexual harrasement in the workplace. 3) Knowledge of how to prevent HIV has improved and in high-burden countries, gender gaps between young women and men (15-24) on this indicator are closing. Nonetheless, gender discrimination and violence limit girls' ability—as compared to young men—to negotiate condom use. 4) Gender inequalities among refugee children and adolescents are also alarming: compared to boys, only half as many refugee girls attend secondary school because of fears for their safety from rape and kidnapping, or lack of proper hygiene facilities.

**Health Clusters and Accountability to Affected Populations: Summary of Feedback from Health Cluster Coordination Teams Working to Ensure Accountability to Affected Population**	WHO	This study aims to capture and share experiences when leading, with cluster partners, emergency responses that have strong and robust accountability systems, through which affected populations can increasingly influence the type, delivery and quality of assistance they receive.	The focus is on the operational guidance on accountability to affected populations (AAP), which was designed to assist Health Cluster Coordination Teams in leading emergency responses that have strong and robust accountability systems, through which affected populations can increasingly influence the type, delivery and quality of assistance they receive.			1) Reproductive health was included in trainings. 2) One of the specific changes made to the respondents' response in order to meet specific needs in light of disaggregated data was sharing SADD for line listing. It helped the planner to plan women's contraceptive programs and helpp other actors for designing their interventions for pregnant or lactating women. 3) Another example is designing messages to promote access to health services or interventions to women, girls, boys and men. 4) Certain activities only target children under 5-years-old, pregnant women and lactating women. 5) Also, specific needs of women of child bearing age, pregnant women or children at different age groups, elderly etc are highlighted and plans made accordingly to address their needs.	1) There was awareness of the need to identify the sectors within affected populations determined by gender and other relevant descriptors for each situation. 2) 50% of the thirteen HSSs reported increasing awareness of Health Cluster Partners on gender, protection and diversity in their cluster and one third expressed that they do not do anything to increasse awareness among partners. 3) Trainings included key gender aspects of training topics, including clinical management of impact of raping, anti-natal care, postnatal care and treatment of severe acute malnutrition cases. 4) Equal opportunity is provided to female health care providers to participate in the training sessions. 5) Several countries are updating their national preparedness plans and AAP, gender an protection are included in some, but not systematically in all. 6) In Iraq all clusters had to incorporate protection, GBV, into their programmatic plan. But there is one specific limitation in that the justice systems do not allow survivors to complain. 7) 53% of respondents reported that Sex and Age Disaggregated Data (SADD) is routinly collected, analysed and used to set a health baseline. 8) Those who ensured SADD, allowed for seperate and confindential discussions with different community groups (including gender and age disaggregated groups) and identifying specific needs and gaps in the response to each sex and age group to ensure responses are underpinned by an understanding of the affected population. 9) 65% of respondents reported that they collaborated with other clusters to investigate and address GBV. 10) 31% of respondents reported performace of the Health Cluster Performance Monitoring (CCPM) excercise with involvement of nationa NGOs and community consultation to review collective strength and weakness against AAP, gender, protection and diversity targets. 11) Recommendations has ben made to partners to incorporate budget lines to support the implementation of AAP, gender, protection and diversity related actions. 12) AAP, gender, protection and diversity related actions are gradually increasing in the field. 13) Monthly meetings with community leaders (including representatives of women and youth) share information about the implementation of projects and receive the complaints and observations of the beneficiaries. 14) Initially women were not consulted and involved in project monitoring and evaluation.
